# HIV Case Management Support Service Is Associated with Improved CD4 Counts of Patients Receiving Care at the Antiretroviral Clinic of Pantang Hospital, Ghana

**DOI:** 10.1155/2017/4697473

**Published:** 2017-09-20

**Authors:** Bismark Sarfo, Naa Ashiley Vanderpuye, Abigail Addison, Peter Nyasulu

**Affiliations:** ^1^Department of Epidemiology and Disease Control, School of Public Health, College of Health Sciences, University of Ghana, Legon, Ghana; ^2^West Africa AIDS Foundation (WAAF), Haatso, Accra, Ghana; ^3^Division of Epidemiology & Biostatistics, School of Public Health, Faculty of Health Sciences, University of the Witwatersrand, Johannesburg, South Africa

## Abstract

**Background:**

Factors associated with individual patient-level management of HIV have received minimal attention in sub-Saharan Africa. This study determined the association between support services and cluster of differentiation 4 (CD4) counts among HIV patients attending ART clinic in Ghana.

**Methodology:**

This was a cross-sectional study involving adults with HIV recruited between 1 August 2014 and 31 January 2015. Data on support services were obtained through a closed-ended personal interview while the CD4 counts data were collected from their medical records. Data were entered into EpiData and analyzed using Stata software.

**Results:**

Of the 201 patients who participated in the study, 67% (129/191) received case management support service. Counseling about how to prevent the spread of HIV (crude odds ratio (cOR) (95% confidence interval (CI)) (2.79 (1.17–6.68)), mental health services (0.2 (0.04–1.00)), and case management support service (2.80 (1.34–5.82))) was associated with improved CD4 counts of 350 cells/mm^3^ or more. After adjusting for counseling about how to prevent the spread of HIV and mental health services, case management support service was significantly associated with CD4 counts of 350 cells/mm^3^ or more (aOR = 2.36 (CI = 1.01–5.49)).

**Conclusion:**

Case management support service for HIV patients receiving ART improves their CD4 counts above 350 cells/mm^3^. Incorporating HIV case management services in ART regimen should be a priority in sub-Saharan Africa.

## 1. Introduction

Although sub-Saharan Africa remains the home to most of new HIV infections [1], there is limited attention observed in terms of care and support provided to persons with HIV/AIDS over and above antiretroviral treatment (ART) programs. Case management support service is a special kind of service model that is usually provided to HIV patients and it consists of the provision of support services to clients and establishing a special relationship and communication between HIV service providers and their clients [2, 3]. This ensures efficient coordination in the provision of medical care and social support services to patients. Ultimately, the goal of case management support services is to effectively engage and retain patients and ensure good adherence to medication and treatment as a whole [4, 5].

In Ghana there is an increase in the number of individuals accessing HIV treatment with over 120,000 people living with HIV/AIDS currently accessing ART [6]. Expanding access to ART calls for reinforcement of effective and efficient care and support services for individuals on treatment. This is crucial for patients' retention and treatment outcomes.

Adequate support services lead to efficient continuum of care from HIV diagnosis through to successful treatment which is central to the monitoring of HIV treatment outcomes. It has been reported that providing support services such as social, physical, and spiritual care is an important part of HIV/AIDS clinical management [7–9].

HIV-infected individuals have increased suffering such as fatigue, fever, pain, and headache [10, 11] and mental health issues [12, 13] whether patients are on ART or not. Successful treatment with positive treatment outcomes requires combination of interventions and antiretroviral therapy together with effective care and support systems. As such there is the need to design strategies and systems to address and manage specific clinical symptoms of HIV-infected individual as a way of maximizing HIV treatment and improve quality of life. The structure of case management support service could differ from one place to another. The support services that are provided at the Pantang ART clinic in Ghana to patients include how to get various laboratory tests performed, keeping up appointments, family issues including children and spousal, and food and transportation.

Studies that examined structural issues such as healthcare system factors and individual level factors relating to the continuum of HIV care, ART use, and engagement and retention in care are limited in sub-Saharan Africa [14].

Loss to follow-up (LTFU) of patients within the HIV/AIDS care continuum has not been well studied in low- and middle-income countries [15] and Ghana is even yet to identify the factors associated with LTFU in HIV-infected populations.

In Ghana, healthcare facilities that provide HIV/AIDS services do not facilitate provision of comprehensive support services such as HIV/AIDS case management, peer group support, medication adherence, and nutritional enhancement among others due to inadequate resources. Meanwhile other studies have demonstrated that the provision of support services such as health insurance and transportation services is associated with retention of care of HIV patients [16].

This study aimed at characterizing relevant care and support services that HIV-infected individuals receive at the Pantang ART clinic, and examine the association between these services and CD4 T cell counts.

## 2. Materials and Methods

### 2.1. Study Site

The study was conducted in the Pantang submunicipality, at Pantang Hospital ART Center, located at the La-Nkwantanang Madina Municipality of the Greater Accra region of Ghana. The Pantang hospital has two major health departments: the general hospital care services department and specialized psychiatry care hospital department. The ART center is attached to the general hospital care services. The Pantang ART center started in 2006 as an HIV testing and counseling unit of the Pantang General Hospital. In 2008, the unit was designated as an ART center to start offering full ART services. Currently the center renders HIV testing and counseling, provision of antiretrovirals (ARVs), Prevention of Mother-to-Child Transmission (PMTCT) services, and community health education on HIV/AIDS services.

There are over 800 HIV/AIDS patients including 20 children receiving ART services at the center presently.

### 2.2. Study Design

Cross-sectional study design was used for this study. Data was collected from patients on ART between August 2014 and January 2015 through medical record abstraction and personal interviews using a closed-ended questionnaire adapted from the Centers for Disease Control and Prevention (CDC), Medical Monitoring Project (MMP) questionnaire. HIV positive patients, 18 years or older, receiving care at the center with the ability to provide informed consent for interview were recruited into the study. Pregnant women were excluded. The study received ethical approval from the Ghana Health Service Ethical Review Committee (GHS-ERC: 17/05/14) and the Noguchi Memorial Institute for Medical Research Ethical Review Committee (NMIMR-IRB CPN-079/13-14) of the University of Ghana. Participants' consent was obtained to publish the findings from this study.

### 2.3. Data Collection Tools

An amended version of the Centers for Disease Control and Prevention (CDC), Medical Monitoring Project (MMP) interview module, which contains questions on sociodemographic characteristics, access to healthcare, support services received by HIV patients, use of antiretroviral drugs, adherence to medication, stigma, CD4 count status and viral load, and a designed data abstraction form were the instruments used for the data collection.

### 2.4. Sampling Procedure and Data Collection

The sample size for this pilot study was estimated using Epi Info 7™. With a population of 800 people living with HIV (PLWH), an estimated ART coverage of 65% at the ART center and at a confidence limit of 5%, 200 participants were estimated for this study.

The participants were selected using the consecutive sampling method. Patients who fell within the eligibility criteria and visited the facility during the study period (August 2014 to January 2015) for their routine care were approached for consent to participate in the study. All who accepted to participate were interviewed. The structured interview questionnaire was used for the collection of personal information and care received by the participants. Data on sociodemographic characteristics, support services received by patients, self-reported adherence to medication, and stigma were collected. Subsequently, patients' medical records were reviewed to validate some of the interviewed responses and also collect data on types of ART medication being used by participants as well as their CD4 counts. CD4 T cell counts were categorized into 350 cells/mm^3^ or below based on the World Health Organization (WHO) guideline (2010 revised in 2013) which recommended the initiation of ART when CD4 T cell counts are less than 350 cells/mm^3^ (WHO 2013). We could not obtain viral load status of participants due to lack of data.

### 2.5. Outcome of Interest

The outcome of interest is CD4 T cell counts of 350 cells/mm^3^ or more after participants enrollment on ART for at least six months. This is based on the World Health Organization (WHO) guideline (2010 revised in 2013) which recommended the initiation of ART when CD4 T cell counts are less than 350 cells/mm^3^ [17].

### 2.6. Exposure Variables

Participants were asked if they received the following support services: case management support service, help remembering to take HIV medicines on time, counseling about how to prevent the spread of HIV, HIV peer group support, mental health service support, drug or alcohol counseling, nutritional service support, and domestic violence support service. Each of these variables was dummy-coded with value 1 indicating they have received the service and a 0 indicating that no such service was received.

### 2.7. Statistical Analysis

Data were entered into EpiData version 3.1. Double entry was done to check for consistency. Entries from the EpiData were then exported into Stata version 13 (Texas, USA) for data cleaning. During the cleaning, all “Don't know” and “Refuse to answer” responses, and a case in which there were no responses, were all treated as missing. Summary statistics were run to determine the mean age and their corresponding standard deviations as well as the generation of frequency tables for the various variables. Chi square test was performed to determine the associations between the various antiretroviral drugs and CD4 cells counts below and above 350 cells/mm^3^. Univariate and multivariable adjusted logistic regression models were performed to distinguish between support services and CD4 counts. *P* value less than 0.05 was considered statistically significant. Variables which had *P* < 0.05 at the univariate analysis were included in the multivariable adjusted model.

## 3. Results

Two hundred and one (*N* = 201) HIV patients participated in the study. The mean age for males and females was 44.8 (SD ± 10.25) and 38.3 (SD ± 9.78) years, respectively (Table 1). Most of the participants were between 31 and 45 years (111/197; 56.3%) and over 51.7% (90/174) of them were married. There were more female participants (152/201; 75.6%) than males (49/201; 24.4%) in the study group (Table 2). Most of the participants had attained junior high school education (89/200; 44.5%). Greater proportion of the participants (121/193; 62.7%) were self-employed (Table 2). For support services that participants had received 12 months prior to the study, the majority (67.5% (129/191)) had received some form of case management for their treatment and counseling (80.6% (154/191)) about how to prevent the spread of HIV. Only 32.1% (62/193) have received nutritional services and 4.1% (8/194) had received mental health services (Table 3).

Over forty-seven percent (89/186) of the respondents were taking* Tenofovir*,* Lamivudine*, and* Efavirenz *(*TDF, 3TC*, and* EFV*, resp.) drug combination regimen (Table 4).

Before enrolling on ART treatment, 47.7% (*N* = 84/176) had their CD4 count above 350 cells/mm^3^ (Table 5) and after enrolling on ART for more than six months, 64.9% (*N* = 96/148) had their most recent CD4 count above 350 cells/mm^3^ (Table 6).

Table 7 presents the association between support services and CD4 count. Counseling about how to prevent the spread of HIV (cOR = 2.79 (CI = 1.17–6.68)), mental health services (cOR = 0.2 (CI = 0.04–1.00)), and case management support service (cOR = 2.80 (CI = 1.34–5.82)) were associated with improved CD4 counts of 350 cells/mm^3^ or more. After adjusting for counseling about how to prevent the spread of HIV and mental health services, case management support service was significantly (aOR = 2.36 (CI = 1.01–5.49)) associated with CD4 counts of 350 cells/mm^3^ or more.

## 4. Discussion

This study has demonstrated that, beyond ART regimen, supporting people living with HIV (PLWH) in managing their associated problems improves their CD4 counts level.

Services that PLWHIV receive play important role in their recovery, and in this study only 32.1% and 4.1%, respectively, have received nutritional and mental health services. Inadequate nutrition has been shown to be linked with the progression of HIV/AIDS disease. It is anticipated that nutritional counseling would be an integral component of support services that HIV/AIDS patients should receive at ART clinics, because it has been recommended that nutritional support should be part of the care provided to HIV-infected patients [18]. Eighty-two percent (82%) of health facilities in Asia-Pacific, sub-Saharan Africa, and the Americas provide nutritional support services for HIV/AIDS patients [19]. Report from other studies from sub-Saharan Africa has also shown high nutritional support services for HIV/AIDS patients [20]. Surprisingly, such services (32.1%) in this Ghana data are inadequate. Furthermore, support services for patients could be different based on the clinical stage of the HIV disease such that, during stage 3, for example, where maintenance could begin once viral load has been controlled, it requires attention such as mental health issues which could influence adherence to medication. The mental health services provided to patients in the current data are also inadequate. The majority of the participants in this Ghana study were on* Tenofovir*,* Lamivudine*, and* Efavirenz *combination therapy. A study conducted in Barcelona, Spain, indicated that a combination regimen boosted the CD4 count and reduces viral load of participants [21].

Combination of ART medication has been recommended for developing countries under the public health approach to antiretroviral therapy by the World Health Organization [22].

Combined ART regimens have been shown to be more effective compared with a single dose regimen [23].

Early diagnosis and initiation of ART based on current treatment guidelines could reduce HIV- related morbidity and mortality [16]. The majority of the participants had first CD4 count test results below 350 cells/mm^3^ before their enrollment on ART. But after at least six months of ART, the CD4 count of most participants improved to above 350 cells/mm^3^, indicating a positive response to treatment. In a study conducted in Rome, it was discovered that initiation of ART at CD4 counts of 350–500 cells/mm^3^ significantly improved the CD4 count of patients and reduced mortality [24].

This study has further shown that there were associations between some of the support services including counseling, mental health, and case management services and improved CD4 counts in patients receiving ART. This association remains strong for supporting patients in managing their associated problems, after controlling for counseling and mental health services. The likelihood of having improved CD4 counts of 350 cells/mm^3^ or more is higher in those who received case management support services in taking care of their HIV conditions compared with those who did not.

During the roll-out of antiretroviral therapy in resource limited countries, emphasis was placed more on capacity building and sustainability of programs such as voluntary counseling and testing, with only minimal attention being paid to patient-level management issues [25]. Patient-level management issues including stigma, pain, fatigue, depression, finances, sleep difficulties, and family cannot be addressed with antiretrovirals.

Apart from case management services which are performed at the discretion of the clinic staff, and counseling patients in preventing HIV transmission, less attention has been paid to patient-level issues. This is common in many ART facilities in sub-Saharan Africa and the low patients' level support services reported in this study are no exception.

At the Pantang ART clinic in this study, the committed staffs use a patient centered approach to delivery of ART services. Clinic staff would inquire from patients personal issues they have whenever they come in for their medications and medical check-ups. Those who have been identified to have issues other than clinical issues are triaged for support within the remit of the staff. Support services include how to get various laboratory tests performed, keeping up appointments, family issues including children and spousal, food, and transportation. These are important support services that improve upon the coping mechanisms of the patients.

These services help in strengthening the health system which impact patients' health. A study conducted in Georgia, USA, demonstrated loss to follow-up of PLWH due to inadequate support services [25] and has recommended that healthcare systems should respond to HIV/AIDS cases with additional supportive services including appointment reminders for patients, case managers, peer-educators, and counselors among others [26]. This Pantang study has shown that, beyond ART regimen, supporting patients in managing their problems improves their CD4 counts level. Possibly, case management support strengthens compliance by patients which in effect makes the antiretrovirals work better. Admittedly, cost could be a factor in incorporating case management into the general ART regime in developing countries. It is therefore important for future studies to determine the cost component of case management in HIV/AIDS intervention in sub-Saharan Africa.

### 4.1. Study Limitation

The most recent CD4 count of some of the participants in this study was recorded in 2013 as they indicated that they could not afford the cost of CD4 test in private laboratories and hospitals. Therefore, the interpretation of the CD4 count must be done with caution. Also the nonavailability of viral load testing is another limitation of the study. Furthermore, since this is a cross-sectional study with consecutive sampling, the outcome cannot be generalized to the entire HIV/AIDS population on ART in Ghana. Nonetheless this study highlights important aspects of HIV/AIDS management in developing countries.

## 5. Conclusion

Patients on ART who received case management support service have improved CD4 count of 350 cells/mm^3^ or more. Incorporating HIV/AIDS case management services in ART regimen should be a priority in sub-Saharan Africa.

## Figures and Tables

**Table 1:** Participants age distribution by sex.

Sex	Mean	Std dev
Male	44.79	10.25
Female	38.34	9.78

**Table 2:** Sociodemographic characteristics of participants.

Variable	Frequency (*N* = 201)	Percentage
*Age (years) *(*N* = 197)		
18–30	34	17.3
31–45	111	56.4
46–65	50	25.4
>65	2	1.0
*Sex *(*N* = 201)		
Male	49	24.4
Female	152	75.6
*Education *(*N* = 200)		
Never attended school	25	12.5
Primary school	39	19.5
Junior high school	89	44.5
Senior high school	35	17.5
Tertiary	12	6.0
*Marital status *(*N* = 174)		
Single	38	21.8
Married	90	51.7
Divorced	15	8.6
Cohabiting	23	13.2
Widowed	8	4.6
*Employment status *(*N* = 193)		
Unemployed	29	15.0
Self-employed	121	62.7
Employed for wages	43	22.3

**Table 3:** Characteristics of support services participants received 12 months prior to the commencement of the study.

Support	Frequency (*N* = 201)	Percentage
*Case management of HIV/AIDS *(*N* = 191)		
No	62	32.5
Yes	129	67.5
*Help remembering to take your HIV medicines on time or correctly *(*N* = 186)		
No	186	73.1
Yes	50	26.9
*Counselling about how to prevent the spread of HIV *(*N* = 191)		
No	37	19.4
Yes	154	80.6
*HIV peer group support *(*N* = 193)		
No	190	98.5
Yes	3	1.5
*Mental health services *(*N* = 194)		
No	186	95.9
Yes	8	4.1
*Drug or alcohol counselling or treatment *(*N* = 194)		
No	161	83.0
Yes	33	17.0
*Domestic violence services *(*N* = 189)		
No	182	96.3
Yes	7	3.7
*Nutritional services *(*N* = 193)		
No	131	67.9
Yes	62	32.1

**Table 4:** Antiretroviral drugs being taken by participants.

Medication	Frequency (*N* = 186)	Percentage
3TC, d4T, NVP	3	1.6
AZT	1	0.5
AZT, 3TC, EFV	26	14.0
AZT, 3TC, NVP	17	9.1
AZT, 3TC, TDF	1	0.5
AZT, TDF, NVP	1	0.5
EFV, 3TC, NVP	1	0.5
Cotrimoxazole	9	4.8
TDF	4	2.2
TDF, 3TC, EFV	89	47.9
TDF, 3TC, NVP	22	11.8
TDF, FTC, EFV	11	5.9
d4T, EFV, TDF	1	0.5

3TC = Lamivudine; d4T = Stavudine; NVP = Nevirapine; AZT = Zidovudine; EFV = Efavirenz; TDF = Tenofovir.

**Table 5:** Participants first CD4 count results before taking antiretroviral medication.

CD4 count (cell/mm^3^)	Frequency (*N* = 176)	Percentage
Below 350	92	52.3
350 and above	84	47.7

**Table 6:** Participants most recent CD4 count results after taking antiretroviral medication for at least 6 months.

CD4 count (cell/mm^3^)	Frequency (*N* = 148)	Percentage
Below 350	52	35.1
350 and above	96	64.9

**Table 7:** Univariate and multivariate analysis of support services participants received (12 months prior to the study), in association with their most recent CD4 count levels.

Support	Unadjusted	*P* value	Adjusted	*P* value
	OR (95% CI)		OR (95% CI)	
*Case management of HIV/AIDS*				
disease (*N* = 191)				
No	1 (ref)		1 (ref)	
Yes	**2.80 (1.34**–**5.82)**	**0.01**	**2.36 (1.01**–**5.49)**	**0.05**
*Help remembering to take HIV medicines on time or correctly*				
No	1 (ref)			
Yes	1.52 (0.66–3.51)	0.32	ND	NA
*Counselling about how to prevent the spread of HIV*				
No	1 (ref)		1 (ref)	
Yes	**2.79 (1.17**–**6.68)**	**0.02**	**1.83 (0.66**–**5.07)**	**0.24**
*HIV peer group support*				
No	1 (ref)			
Yes	1.07 (0.09–12.07)	0.96	ND	NA
*Mental health services*				
No	1 (ref)		**1 **(ref)	
Yes	**0.2 (0.04**–**1.00)**	**0.05**	**0.28 (0.05**–**1.70)**	**0.16**
*Drug or alcohol counselling or treatment*				
No	1 (ref)			
Yes	0.76 (0.31–1.85)	0.54	ND	NA
*Domestic violence services*				
No	1 (ref)			
Yes	2.24 (0.24–20.61)	0.48	ND	NA
*Nutritional services*				
No	1 (ref)			
Yes	0.79 (0.38–1.67)	0.54	ND	NA

ND = not determined; NA = not applicable.

## Additional Points

*Availability of Data and Material.* The datasets for the current study are available from the corresponding author on reasonable request.
